# Urolithin A: a multi-target therapeutic candidate derived from the gut microbiota for obesity and metabolic dysfunction

**DOI:** 10.3389/fendo.2026.1786776

**Published:** 2026-03-10

**Authors:** Chang Liu, Mingxing Sun, Zhiping Zhao, Ying Yang, Yikun Yang, Yujun Zhao, Rui Zhang, Xiuwei Du, Xue Liu, Shuying Ran, Yanfang Wang, Xiaogang Pang

**Affiliations:** 1 Experimental Center, Shandong University of Traditional Chinese Medicine, Jinan, China; 2 College of Traditional Chinese Medicine, Shandong University of Traditional Chinese Medicine, Jinan, China; 3 School of Nursing, Shandong University of Traditional Chinese Medicine, Jinan, China; 4 Innovative Institute of Chinese Medicine and Pharmacy, Shandong University of Traditional Chinese Medicine, Jinan, China; 5 College of Pharmacy, Shandong University of Traditional Chinese Medicine, Jinan, China; 6 Medical College, Shandong University of Traditional Chinese Medicine, Jinan, China

**Keywords:** adipose tissue browning, gut microbiota metabolism, obesity, tannins, Urolithin A (UroA)

## Abstract

This review comprehensively examines the role and mechanisms of Urolithin A (UroA), a gut microbial metabolite derived from dietary ellagitannins (ETs), in ameliorating obesity and related metabolic disorders. The *in vivo* production of UroA is strictly dependent on specific gut microbiota, and the substantial inter-individual variation in this metabolic capacity (UM phenotype) directly influences population responsiveness to ETs-rich dietary interventions. Mechanistically, UroA acts through multiple coordinated pathways: it activates thermogenesis in brown and beige adipose tissue to promote energy expenditure; bidirectionally regulates lipid metabolism by enhancing fatty acid oxidation while suppressing lipogenesis; remodels the immune microenvironment by polarizing macrophages toward the anti-inflammatory M2-like phenotype to alleviate chronic inflammation; and modulates gut microbiota composition at multiple taxonomic levels and regulates microbial tryptophan metabolism, alongside enhancing intestinal barrier integrity. These integrated effects collectively improve systemic insulin sensitivity, glucose homeostasis, and reduce lipid accumulation. Although preclinical evidence is robust, its efficacy in humans requires further validation through large-scale clinical trials. In summary, UroA represents a pivotal active molecule within the “diet-microbiota-host” interaction axis, offering a novel scientific rationale and a potential target for developing personalized nutritional strategies against obesity and other metabolic diseases.

## 1 Introduction

Obesity is defined by the World Health Organization as a physiological condition characterized by a body mass index (BMI) of ≥30 kg/m² (1). BMI is calculated as weight in kilograms divided by the square of height in meters. However, this criterion can be misleading for individuals with significantly increased muscle mass, as it fails to distinguish between the contributions of fat and lean tissue to body weight. In contrast, body fat percentage (BF), with diagnostic thresholds of ≥25% for men and ≥35% for women, provides a more accurate reflection of adiposity (2). Waist circumference (WC), a measure of central obesity, has Asian-specific cut-offs of ≥90 cm for men and ≥80 cm for women (3). Evidence-based medicine indicates that WC, either alone or in combination with BMI, correlates significantly more strongly with the risk of metabolic syndrome and cardiovascular disease than BMI alone (4). Notably, reduced gut microbiota alpha-diversity is common in obesity. Based on this, the Gut Microbiome Health Index (GMHI) has been proposed; it compares fecal metagenomic species abundance between healthy and obese individuals to construct a disease prediction model independent of traditional clinical parameters, offering a novel biomarker for obesity diagnosis (5).

As a global public health crisis, the prevalence of obesity continues to rise and is closely linked to serious complications such as metabolic syndrome, cardiovascular disease, and type 2 diabetes. According to the International Diabetes Federation’s Diabetes Atlas, 589 million adults aged 20–79 were living with diabetes in 2024. Type 2 diabetes accounts for over 90% of all cases and is frequently associated with obesity (6, 7). Disorders in lipid metabolism are initially reflected in altered blood lipid profiles, marked by significantly elevated levels of triglycerides (TG), total cholesterol (TC), and low-density lipoprotein cholesterol (LDL-C), alongside decreased high-density lipoprotein cholesterol (HDL-C) (8). Dysfunctional adipose tissue is characterized by adipocyte hypertrophy, inflammatory infiltration, fibrosis, and mitochondrial dysfunction. Metabolic disorders like obesity and type 2 diabetes, driven by such tissue dysfunction, pose a major public health challenge. While traditional strategies—lifestyle modification, pharmaceuticals, and surgery—can partially alleviate symptoms, their long-term efficacy is limited and they are often accompanied by side effects. Consequently, exploring safe interventions based on natural products has become a key research focus.

Tannins, secondary plant metabolites, originally functioned as a defense against herbivores and pathogens (9). Clinical studies show that daily intake of 150 mg of low-molecular-weight procyanidins can reduce systolic blood pressure by 6.36 mmHg (p=0.014) and increase HDL-C levels by 14.06% (p=0.012) in hypertensive patients (10). Supplementation with ellagic acid (EA) has been shown to mitigate metabolic alterations and characteristic structural/functional changes in visceral organs (heart and liver) induced by a high-fat diet (11). Further research indicates that urolithins, such as Urolithin A (UroA), are the active metabolites responsible for the biological effects of ellagitannins (ETs) and EA. The first natural compound in this family, discovered in 1949 by Tomas and named “Castoreum Pigment,” was isolated from the dried scent glands (castoreum) of beavers (Castor fiber) (12). In 1964, a compound isolated from the kidney stones of sheep with alfalfa stone disease was named Urolithin A. In 1980, Doyle and Griffiths demonstrated that rat gut microbiota could convert EA to UroA both *in vitro* and *in vivo*, whereas germ-free rats fed EA produced no such metabolite. Subsequent studies confirmed that both rats and humans generate UroA after consuming pomegranate extract (ETs). Given that urolithins are absorbed much more efficiently than their parent ETs and EA, they are considered the likely mediators of the health benefits associated with ET-rich foods like pomegranates, berries, walnuts, other nuts, many tropical fruits, medicinal plants, and herbal teas. UroA has now been convincingly shown to significantly inhibit triglyceride accumulation in both adipocytes and hepatocytes (13).

Recent studies indicate that a high-fat diet can markedly alter gut microbiota composition, characterized by features such as decreased Bacteroidetes and expanded Firmicutes abundance. This microbial dysbiosis exacerbates obesity by affecting energy metabolism and inflammatory pathways (14). Therefore, targeting the restoration of gut microbiota homeostasis has emerged as a vital direction for obesity intervention strategies.

This review focuses on UroA, the terminal metabolite of tannin biotransformation. We aim to systematically elucidate its multi-target regulatory mechanisms in obesity. We will first summarize the natural sources and structural classification of tannins, as well as the gut microbiota-dependent metabolic pathway leading to UroA. Next, we will outline the molecular mechanisms underlying obesity pathogenesis, with an emphasis on the interplay between adipose tissue homeostasis disruption and gut microbiota dysbiosis. Finally, we will delve into the potential mechanisms through which UroA counteracts obesity, including activation of adipose tissue browning, promotion of lipolysis, improvement of insulin sensitivity, and modulation of microbiota-host crosstalk. This review seeks to provide a theoretical foundation and innovative perspectives for the advanced development of natural polyphenol resources and the precise intervention of metabolic diseases.

## 2 Source and structure of ellagitannins

Tannins are a common class of polyphenolic secondary metabolites in higher plants, widely distributed in tissues such as bark, fruit, leaves, and seeds. Based on their chemical structure, tannins are primarily classified into two fundamental types: hydrolyzable tannins and condensed tannins (15). Hydrolyzable tannins can be fractionated via hydrolysis using hot water or tannase enzymes, whereas condensed tannins refer to non-hydrolyzable oligomers and polymers of proanthocyanidins. Another group, often termed complex tannins, consists of non-classical structures where catechin units are linked to glycosides via C-C bonds, conferring only partial hydrolyzability. This led to the subsequent proposal of a tripartite classification system: condensed tannins, hydrolyzable tannins, and complex tannins (16). In recent years, with advances in structural elucidation, a more taxonomically informative quadripartite system has gained acceptance, further categorizing tannins into condensed tannins, complex tannins, gallotannins, and ellagitannins (16). Within this framework, both ellagitannins (ETs) and gallotannins (GTs) traditionally fall under the broader category of hydrolyzable tannins.

Ellagitannins are widely distributed in nature. They are found predominantly in berries (e.g., strawberries, Rubus species), pomegranates, muscat grapes, walnuts, almonds, as well as in cereals and tea (17). Representative concentration ranges include 2020–2660 mg/L of punicalagin in pomegranate juice, 130.0 mg/kg of punicalin in pomegranate peel, and up to 16 mg/g of pedunculagin in walnuts (18).

The molecular weight of ETs significantly influences their bioavailability. Experimental data show that the transfer efficiency of ETs into fruit juice decreases by more than an order of magnitude as their molecular weight increases from 1568 Da to 2805 Da (19). This molecular sieving effect causes higher molecular weight ETs to be retained more readily in pomace. For instance, despite their water-soluble nature, approximately 80% of ETs remain in the raspberry pomace matrix after pressing (19). Consequently, consuming whole fresh fruit is a more effective way to obtain ETs compared to drinking juice alone.

It is also noteworthy that ET content varies among different cultivars of the same fruit. In pomegranates, for example, significant differences in ET levels exist among various germplasms, with wild, sour-tasting varieties typically exhibiting higher total polyphenol and punicalagin content (20). Tissue-specific analysis further reveals that punicalagin accumulates most prominently in the placental tissue of pomegranates (21). Additionally, developmental studies confirm that the content of total polyphenols, total flavonoids, total flavanols, and punicalagin in various pomegranate organs declines throughout development, with a corresponding decrease in antioxidant activity (21).

## 3 Generation of Urolithin A: gut microbiota-dependent conversion and individual variation

### 3.1 Gut microbiota-dependent biotransformation: from ellagitannins to Urolithin A

Urolithin A (UA) belongs to the dibenzo[b,d]pyran-6-one class of compounds, characterized by a core structure in which two benzene rings are connected via a pyranone ring. Chemically, these molecules are also described as benzocoumarins or dibenzopyrones. UA is not present as such in foods; rather, it is a microbial metabolite produced by the gut microbiota from dietary precursors such as ellagitannins (e.g., punicalagin in pomegranate) or ellagic acid.

**Figure 1:**
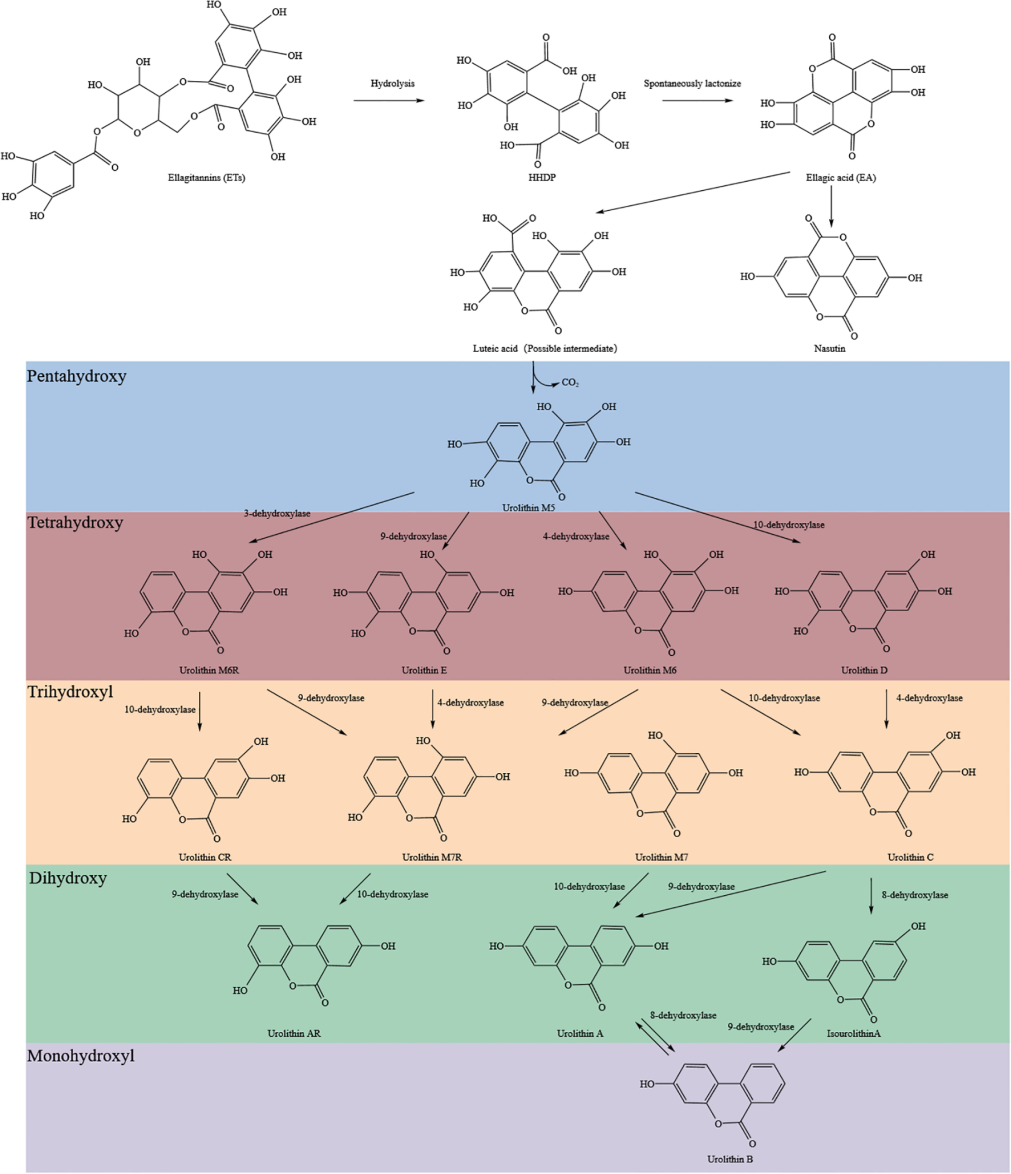
Chemical pathway of ellagitannins metabolism by gut microbiota. Ellagitannins (ETs) are hydrolyzed to hexahydroxydiphenic acid (HHDP) and spontaneously lactonized to ellagic acid (EA), which is sequentially converted by gut microbiota to various urolithins through dehydroxylation reactions.

Ellagitannins (ETs) are condensed products derived from the natural polyphenol ellagic acid (EA). Studies show that ETs, like punicalagin, undergo acid- or base-mediated hydrolysis in the stomach and small intestine to yield hexahydroxydiphenic acid (HHDP), which spontaneously lactonizes to form EA (22). As a highly polar, high-molecular-weight compound that does not comply with Lipinski’s Rule of Five (23). EA is poorly absorbed directly from the gastrointestinal tract. Only trace amounts are taken up by small intestinal epithelial cells. The majority of EA reaches the colon, where it is metabolized by the gut microbiota into hydroxylated 6H-dibenzo[b,d]pyran-6-one derivatives known as urolithins (Uros) (24). The detailed conversion pathway from ETs to urolithins is summarized in **Figure 1** (22, 25, 26).

To date, nine natural urolithins have been identified: Uro A–E, M5, M6, M7, and iso-Uro A. Based on the number of hydroxyl substituents, they can be divided into five categories: pentahydroxy (Uro-M5), tetrahydroxy (Uro-D/M6/E/M6R), trihydroxy (Uro-C/M7/M7R/CR), dihydroxy (Uro-A/iso-Uro A/AR), and monohydroxy (Uro-B).

Owing to their lipophilic nature, Uros readily cross the intestinal epithelium and enter systemic circulation; however, their bioavailability is highly dependent on individual gut microbiota composition. This variability is largely explained by distinct urolithin metabotypes (UMs). Current research classifies individuals into three UM subgroups: UM-A, capable of producing UroA and its conjugates; UM-B, which yields UroA, isoUroA, and UroB; and UM-0, in whom no urolithin metabolites are detected (12). This metabolic stratification significantly contributes to the broad concentration ranges observed in biological fluids.

Upon absorption, urolithins undergo extensive phase II metabolism in the liver, forming glucuronide and sulfate conjugates. Consequently, conjugated forms predominate in systemic circulation, with free (unconjugated) urolithins present at trace levels (27, 28). Total plasma urolithin concentrations typically range from 0.003 to 5.2 μM, with urinary levels reaching up to 50 μM; however, these values are highly variable and depend on the metabotype, dose, and ellagitannin source (27). In a first-in-human trial involving healthy elderly subjects, both single and repeated oral administration of urolithin A (UA) at daily doses of 250–1000 mg over 28 days produced dose-dependent plasma concentrations. Peak concentrations occurred approximately 6 h post-dose (Tmax), and the elimination half-life was around 24 h (27, 29). These pharmacokinetic parameters are summarized in **Table 1**.

**Table 1:** Pharmacokinetic parameters of urolithin A.

Parameter	Value/Range	Unit	Conditions/Notes	Reference
Tmax (Time to Cmax)	6	h	Single oral dose 250–500 mg	(171)
Absolute Bioavailability (F)	3.8	%	PBPK model prediction, oral administration	(172)
Fecal Excretion	96.2	%	Within first 24 h post-dose	(172)
Cmax (Parent Compound)	4-7	nM	Dose range 250–1000 mg (dose-proportional)	(171)
Cmax-Uro-A glucuronide	1500-3000	nM	Major circulating metabolite	(171)
Cmax-Uro-A sulfate	200-400	nM	Minor circulating metabolite	(171)
Relative Exposure (*vs* Pomegranate Juice)	>6-fold	ratio	iAUC of UA-glucuronide: 500 mg UA *vs* pomegranate juice	(173)
Oil-Water Partition Coefficient (Log P)	2.11 ± 0.087	–	Experimental (shake-flask method)	(172)
Maximum Solubility	13.0 ± 0.57	µM	Phosphate buffer (pH 7.4), 37 °C	(172)
Muscle Tissue Concentration	0.9	ng/g	Skeletal muscle, 8 h post single 2000 mg oral dose	(174)
Blood Cell/Plasma Ratio	<1.0	–	Distribution into blood cells	(174)
Primary Metabolic Pathway	Phase‐II metabolism	–	Glucuronidation and sulfation	(174)
Dominant Metabolite	Uro-A Glucuronide	–	Predominant circulating form	(173)
Enzyme Kinetics Km (Liver)	24.5	µM	Hepatic S9 fraction	(172)
Enzyme Kinetics Km (Intestine)	14.4	µM	Intestinal S9 fraction	(172)
Enzyme Kinetics Vmax (Liver)	1.7	nmol min^−1^ mg^−1^	Hepatic S9 protein	(172)
Enzyme Kinetics Vmax (Intestine)	3.6	nmol min^−1^ mg^−1^	Intestinal S9 protein	(172)
Half-life (t1/2) - Parent	17-22	h	Single oral dose	(171)
Half-life (t1/2) - Glucuronide	17-22	h	Similar to parent compound	(171)
Half-life (t1/2) - Sulfate	25-58	h	Longer than glucuronide	(171)
Elimination Time (Complete)	72-96	h	All forms eliminated from circulation	(171)
Steady-State Concentration	400-600	ng/mL	Total Uro-A (parent + metabolites), range across 250–1000 mg doses	(171)

Current evidence suggests that the biological activity attributed to EA may largely originate from its urolithin metabolites (30). However, the high pharmacokinetic variability (0.003–5.2 μM plasma concentration range), the predominance of conjugated forms with potentially different biological activities compared to free urolithins, and the significant proportion of non-producers (UM-0, ~10%) should be considered when interpreting systemic effects (12, 27).

### 3.2 Interindividual variability and the UM−0 metabotype: implications for clinical translation of Urolithin A

Cross-population studies reveal notable variation in UM distribution. Among healthy Spanish adults, the prevalence of UM-A, UM-B, and UM-0 is reported as 55%, 35%, and 10%, respectively (31). Corresponding figures are 54.3%, 28.6%, and 17.1% in a Brazilian cohort (32), and 54.3%, 31.4%, and 14.3% in a group of Chinese adolescents (33). Interestingly, a study of free-living, healthy Americans aged 18–80 found that only about 40% were capable of producing UroA (34). Despite geographical, cultural, and ethnic differences, cross-continental comparisons show a consistent association between specific UM profiles and distinct gut microbial communities, further underscoring the significant interindividual variation in EA metabolism. Based on its safety profile, the U.S. Food and Drug Administration (FDA) granted Urolithin A (UroA) “Generally Recognized As Safe” (GRAS) status in 2018 and approved its use as a dietary supplement to support muscle health in aging adults (18, 22). But the real bottleneck isn’t the molecule itself—it’s the microbiome.

The composition of the gut microbiota is a key determinant of UM phenotype, with each UM associated with a characteristic microbial signature (17). This explains why consuming ET-rich foods does not guarantee increased UroA bioavailability, as its production strictly depends on the presence of specific gut bacteria (35). Microorganisms identified as involved in the conversion of EA to UroA include species from the genera *Gordonibacter* (*G. pamelaeae, G. urolithinfaciens*) *(*36), *Clostridium coccoides (*37), *Bifidobacterium pseudocatenulatum (*38), *Enterococcus faecium (*39), *Streptococcus thermophilus (*40), and *Lactobacillus plantarum (*41).

Emerging evidence indicates that becoming a “UroA producer” depends not only on harboring specific microbes but also on host physiological factors like age and body mass (42). Urolithin excretion levels show marked interindividual variation, are suppressed during diarrheal episodes, but appear unrelated to sex or general nutritional status (32). Furthermore, UM distribution exhibits age-dependent patterns: the proportion of UM-0 individuals remains relatively stable (10.4% ± 1.9%) across ages 5 to 90, while the prevalence of UM-B increases with age, accompanied by a decrease in UM-A. This shift is most pronounced between ages 20 and 40, stabilizing after 41–50 (42). The net effect of aging is a reduction in UroA production and an increase in UroB, resulting in a lower UroA/UroB ratio. The strong correlation between EA metabotype and age suggests that the metabolic function of the involved microbiota may itself be subject to developmental regulation (42).

The existence of UM-0 individuals—who cannot produce UroA from dietary ETs—poses a significant challenge for the universal recommendation of ET-rich diets as a metabolic health strategy. As the population data above illustrate, the proportion of non-producers ranges from approximately 10% to 17% in the cohorts examined, and may be substantially higher in certain settings (32, 43–45). This metabolic stratification creates a “responder gap” where a considerable proportion of the population cannot derive UroA-mediated benefits from consuming ET-rich foods such as pomegranates, berries, or walnuts.

This variability necessitates a personalized nutrition approach rather than a one-size-fits-all dietary recommendation. For UM-0 individuals, alternative strategies must be considered: (1) direct supplementation with synthetic UroA, which bypasses gut microbiota dependency and has been shown to achieve consistent plasma concentrations across populations (29); (2) probiotic interventions using UroA-producing bacterial strains such as Gordonibacter urolithinfaciens or Bifidobacterium pseudocatenulatum to colonize the gut and restore metabolic capacity (38, 46, 47); or (3) combination therapies pairing ET-rich foods with prebiotics that selectively enhance UroA-producing taxa. Notably, the correlation between UM phenotype and age/obesity status suggests that metabolic capacity is dynamic—UM-0 may represent a modifiable state rather than a fixed trait, offering opportunities for microbiome-targeted interventions (42).

Additionally, several studies confirm that the prevalence of the UM-B phenotype is significantly higher in overweight/obese individuals compared to those with normal weight (p<0.05), while UM-A shows an inverse trend (32, 33, 42). This pattern hints that the bacterial consortia responsible for converting ETs/EA to UroA may play a role in regulating host lipid metabolism. Accumulating evidence positions UroA as a potential modulator in the pathophysiology of obesity-related metabolic disorders. Its mechanisms of action, which include improving the function of metabolic tissues to ameliorate conditions like diabetes and obesity, have been substantiated across multiple studies (48–51). These insights have established UroA as a prominent focus of research in the field of metabolic disease intervention.

## 4 Pathophysiological basis of obesity

### 4.1 Functional heterogeneity of adipose tissue and energy metabolism

**Figure 2:**
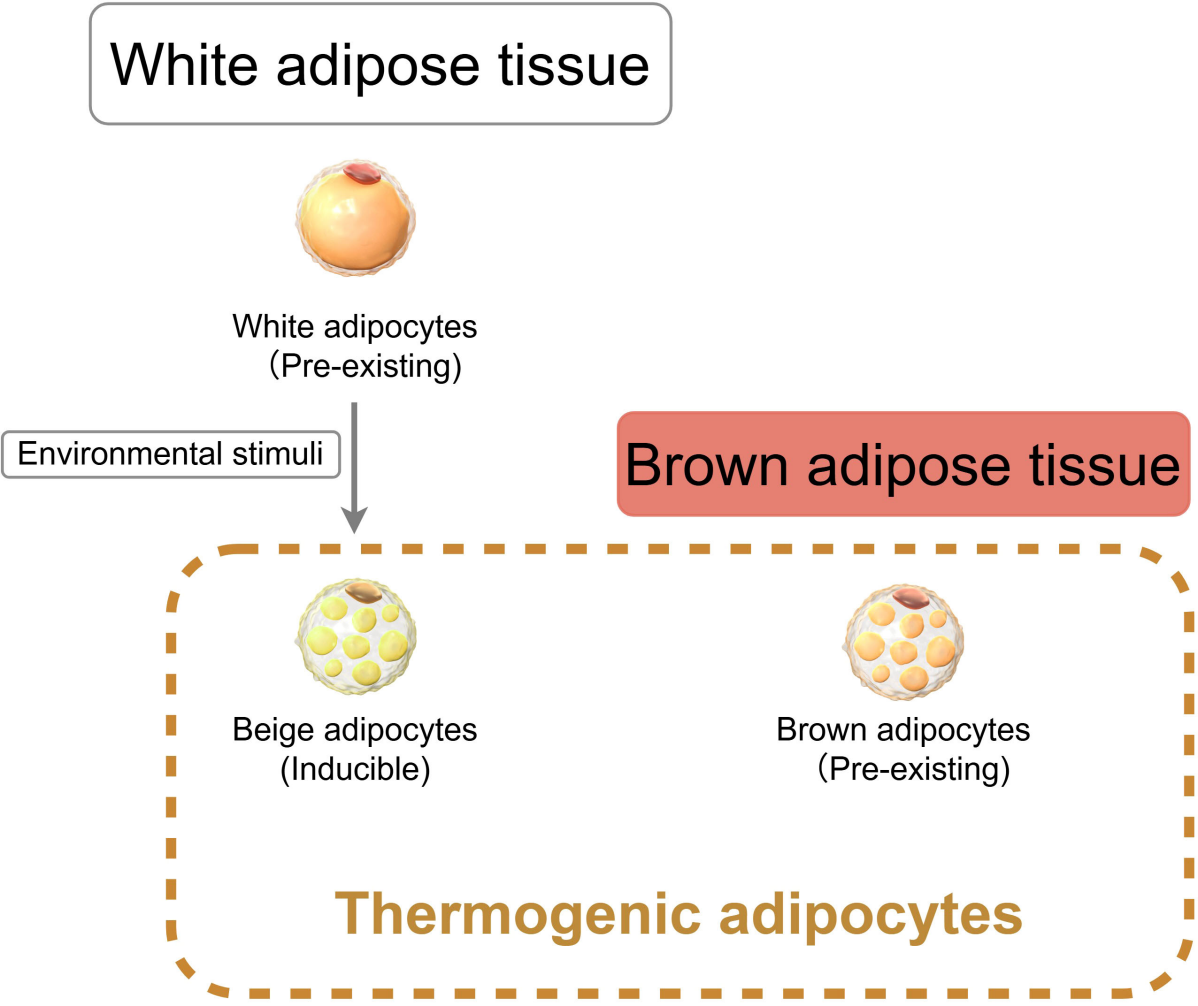
Functional heterogeneity of adipose tissue. White adipose tissue (WAT) contains pre-existing white adipocytes that can transdifferentiate into beige adipocytes under environmental stimuli. Brown adipose tissue (BAT) contains pre-existing brown adipocytes. Beige and brown adipocytes are collectively termed thermogenic adipocytes due to their capacity for heat production.

Mammalian adipose tissue is primarily categorized into white adipose tissue (WAT) and brown adipose tissue (BAT) (**Figure 2**). The metabolic dysregulation characteristic of obesity stems from an imbalance between energy storage and expenditure, driven by the pathological expansion and ectopic deposition of WAT coupled with impaired thermogenic function in brown/beige fat.

WAT is composed of white adipocytes containing a single, large lipid droplet. It is found mainly in subcutaneous depots (e.g., inguinal WAT, iWAT) and surrounding visceral organs (e.g., epididymal WAT, eWAT). These cells contain few mitochondria and store excess energy as triglycerides within their lipid droplets. Under certain environmental stimuli, such as chronic cold exposure or β3-adrenergic receptor activation, a subpopulation of white adipocytes can undergo a metabolic adaptation known as “browning,” transdifferentiating into beige adipocytes (52). Notably, iWAT demonstrates a greater potential for browning compared to eWAT, owing to its smaller adipocyte size and enhanced differentiation capacity (53).

BAT is principally located in the interscapular, cervical, and perirenal regions. Its defining brown adipocytes are characterized by multilocular lipid droplets and a high density of mitochondria. Both beige and brown adipocytes express uncoupling protein 1 (UCP1), a mitochondrial inner membrane protein that dissipates the proton gradient to release chemical energy as heat (54). UCP1 and PGC-1α are hallmark thermogenic genes in BAT. Given their shared capacity for heat production, beige and brown adipocytes are collectively termed thermogenic adipocytes.

Functioning as a significant endocrine organ, WAT secretes adipokines (e.g., adiponectin, RBP4, TNF-α) that play crucial roles in regulating glucose metabolism and inflammation (55). Clinical evidence links abnormal white fat distribution to reproductive endocrine disorders; lipodystrophy can lead to reproductive dysfunction, whereas excessive fat accumulation is associated with precocious puberty (56). Conversely, thermogenic adipose tissue secretes a multitude of peptides and small-molecule metabolites, collectively termed batokines, which act locally (via autocrine/paracrine signaling) or distally (via endocrine signaling) (57). Through these batokines, thermogenic fat exerts both local and systemic control. For instance, factors like BMP8B mediate local thermogenesis and vascular remodeling, while circulating factors such as neuregulin 4 (NRG4) can modulate hepatic lipid metabolism (57, 58).

In obesity, excessive fat accumulation leads to enhanced lipolysis, the hydrolysis of triglycerides into glycerol and free fatty acids (FFAs) (59). The resulting overabundance of FFAs activates inflammatory pathways, induces oxidative stress, and can trigger cellular dysfunction and apoptosis. Obesity is, notably, a key driver of metabolic syndrome, which encompasses central adiposity (increased waist circumference), hypertriglyceridemia, low high-density lipoprotein cholesterol (HDL-C) levels, hypertension, and hyperglycemia.

Clinical research indicates that among individuals exhibiting three or more features of metabolic syndrome, plasma levels of urolithin A glucuronide show a significant inverse correlation with abdominal obesity as measured by waist circumference. In both plasma and urine, UroA is found primarily in its glucuronidated form. The maximum reported concentrations are 0.024–35 μM for UroA glucuronide in human plasma and up to 100 μM in urine (12). The non-glycosylated (aglycone) form of urolithin is present at lower concentrations—approximately 0.005 μM in plasma and 10 μM in urine. Further mechanistic studies suggest that UroA’s bioactivity is independent of its specific metabolic form (glucuronidated, aglycone, or sulfated). The finding that only the free aglycone form is detected in peripheral tissues implies that glucuronide conjugates must undergo hydrolysis to exert their physiological effects (60).

### 4.2 Multifactorial mechanisms in obesity pathogenesis

#### 4.2.1 Genetic susceptibility and environmental factors

As a complex metabolic disease, obesity results from the interplay between genetic predisposition and environmental factors. Inherited mechanisms primarily involve genetic polymorphisms (including epigenetic modifications like DNA methylation and non-coding RNA) and gut microbiome features. Such genetically influenced obesity accounts for 40-70% of all cases (61, 62). Notably, the genetic contribution can reach 80% in individuals who develop severe obesity before the age of 6 (63). While most obesity is polygenic and influenced by the environment, 2-5% of severe childhood obesity cases are attributed to monogenic mutations, with inactivating mutations in the *melanocortin 4 receptor (MC4R)* gene being the most common (64). Both heterozygous and homozygous *MC4R* mutations can cause obesity, accompanied by hyperphagia, hyperinsulinemia, and accelerated linear growth. These mutations primarily disrupt the leptin-melanocortin pathway (LMP), leading to early-onset, severe obesity (62).

**Figure 3:**
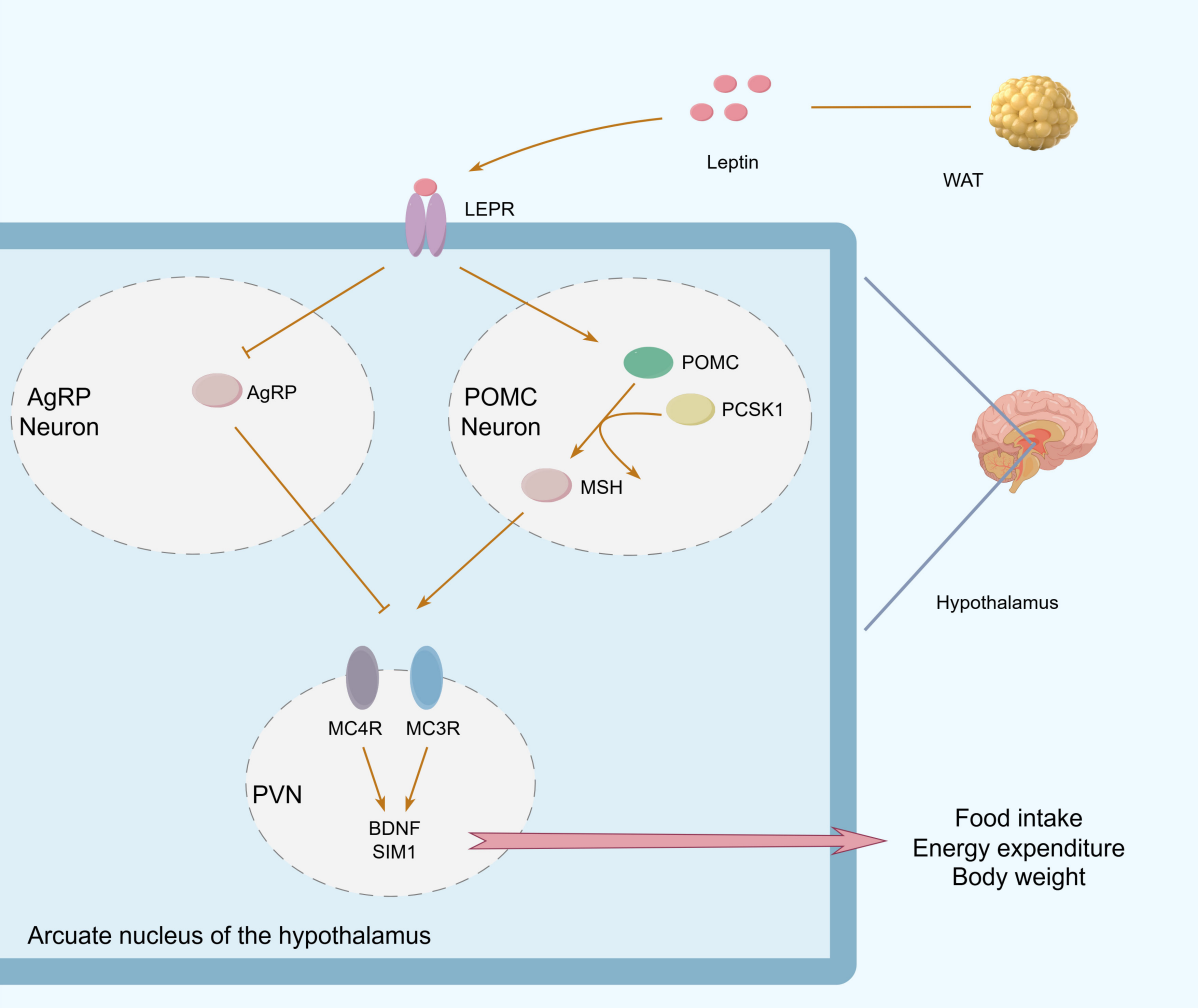
Leptin signaling in the hypothalamic arcuate nucleus. Leptin from WAT activates POMC neurons and inhibits AgRP neurons in the arcuate nucleus, regulating food intake, energy expenditure, and body weight through MC3R/MC4R signaling.

At the molecular level, leptin activates POMC neurons in the hypothalamus, stimulating the synthesis of proopiomelanocortin (POMC). This precursor protein is cleaved to produce hormones like adrenocorticotropic hormone (ACTH) and α-melanocyte-stimulating hormone (α-MSH). α-MSH binds to melanocortin receptors (MC3R/MC4R) in the arcuate nucleus, mediating appetite suppression and increased energy expenditure. In contrast, leptin’s stimulation of AgRP neurons suppresses the secretion of agouti-related peptide (AgRP). This neuropeptide acts as an endogenous antagonist of MC3/4R, blocking receptor activation and thereby promoting feeding behavior (65) (**Figure 3**). It is now established that AgRP signaling not only promotes weight gain by antagonizing MC3/4R but also creates a dynamic balance with POMC neurons, together forming the central melanocortin system (CMS) (66). Beyond this, α-MSH and AgRP can regulate neural circuits through both MC3R-dependent and independent pathways.

Genome-wide association studies (GWAS) and their meta-analyses have so far identified over 750 obesity-associated loci, encompassing more than 3000 single nucleotide polymorphisms (SNPs) (67–73). Among these, the LMP and insulin signaling pathways are recognized as core regulatory networks. Furthermore, genes such as *FTO* (fat mass and obesity-associated), *UCP* (uncoupling protein), and *ANKK1* (ankyrin repeat and kinase domain containing 1) have been found to significantly influence energy homeostasis and thermogenesis, even though they are not part of classic metabolic pathways (74).

**Figure 4:**
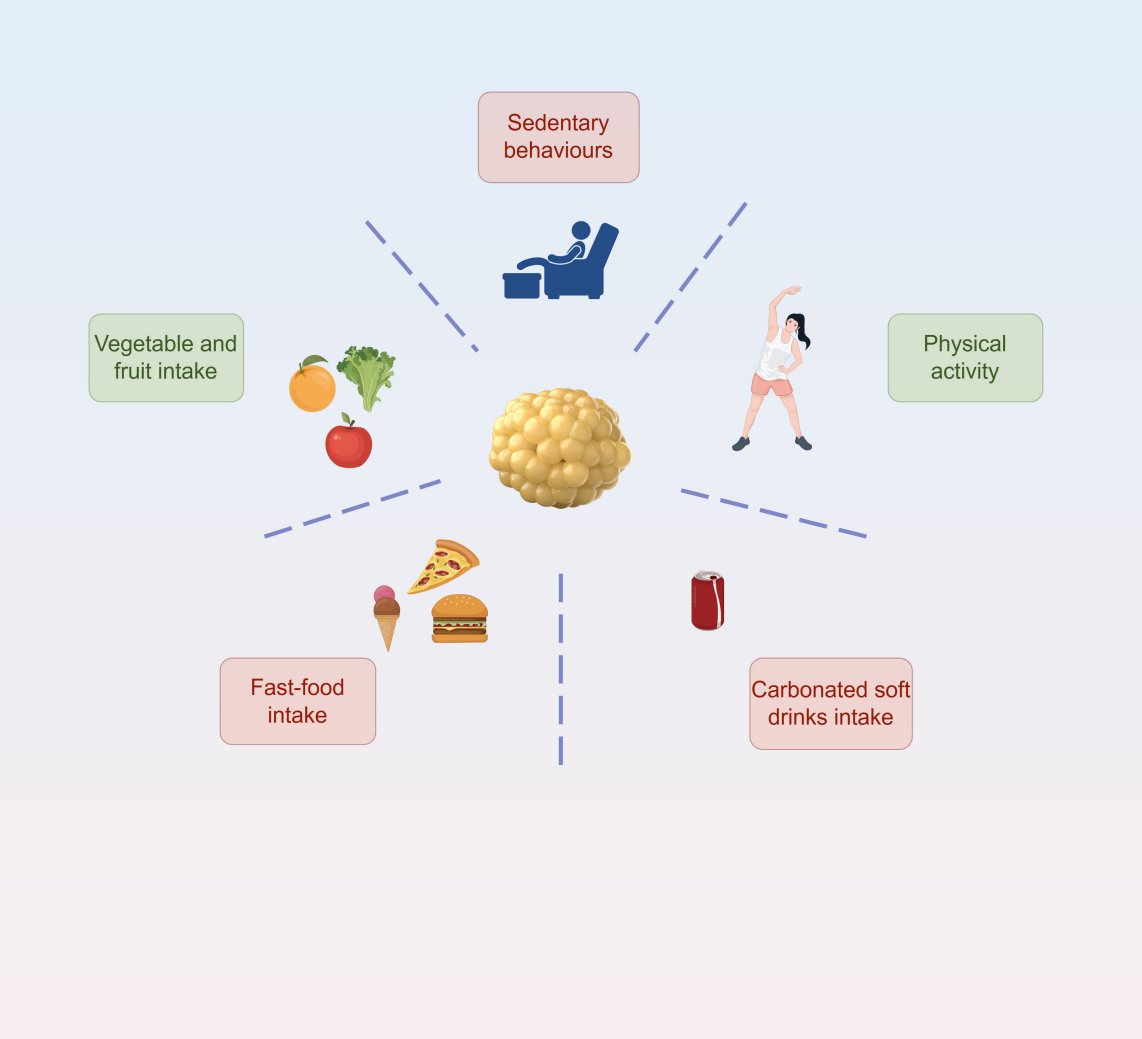
Environmental and behavioral factors influencing obesity. The diagram illustrates the impact of sedentary behaviors, physical activity, dietary patterns (vegetable/fruit intake vs. fast-food and carbonated soft drinks intake) on adipose tissue and obesity development.

Beyond genetic predisposition, the development of obesity is profoundly shaped by environmental and behavioral factors, including dietary patterns, physical activity levels, and technology use (75)(**Figure 4**). Unhealthy diets and lifestyles are strongly linked to overweight and obesity—think frequent fast food and sugary drink consumption, coupled with low physical activity and high amounts of sedentary behavior (76). Certain built environment features, like better street connectivity, higher residential density, and greater access to green spaces, are associated with lower obesity rates. These features encourage physical activity while discouraging sedentary habits. Similarly, easier access to food outlets offering healthy options, such as fruit and vegetable markets, is linked to lower obesity prevalence, as it promotes healthier eating behaviors (77). For example, adolescents who eat vegetables at least twice daily have a 22% lower risk of being overweight and a 17% lower risk of obesity compared to those who don’t consume vegetables daily (76).

#### 4.2.2 Molecular mechanisms linking gut microbiota dysbiosis to obesity

The human gut microbiota constitutes a complex ecosystem, with approximately 10^14^ bacterial cells per gram of colonic content encompassing 400–500 different species. A healthy individual typically harbors around 195 bacterial strains, about 101 of which form the core constituents of their fecal microbiota (78). In recent years, a growing body of research has underscored the pivotal role of the gut microbiota in obesity and related metabolic disorders. Alterations in microbial composition and function can profoundly influence host energy metabolism and immune responses, thereby promoting the development of obesity and insulin resistance (79, 80). Furthermore, the gut microbiota directly contributes to obesity pathogenesis by modulating energy harvest and fat storage mechanisms (81).

Investigations into the link between gut dysbiosis and obesity originated from the hypothesis that the microbiome acts as an environmental factor in weight gain. The first crucial evidence came from germ-free mouse transplantation studies, which demonstrated that gut microbes directly promote host fat accumulation (82). Subsequent research focused on the balance between two dominant bacterial phyla—Firmicutes and Bacteroidetes. Early animal and human studies suggested that obesity might be associated with an increased relative abundance of Firmicutes and a decrease in Bacteroidetes, leading to a higher Firmicutes/Bacteroidetes (F/B) ratio. This shift was thought to enhance the energy harvest from diet (83–86). However, this finding has not been consistent; several later studies reported no significant change or even a decrease in the F/B ratio, indicating that the relationship between gut microbiota and obesity is likely more complex (87–89).

Obesity has also been linked to changes in specific bacterial taxa. For instance, the abundance of *Christensenellaceae* and *Akkermansia muciniphila* correlates positively with weight loss (90, 91). The effects of traditional probiotic genera like *Lactobacillus* and *Bifidobacterium* appear to be species- or strain-specific, with different species within the same genus sometimes exerting opposite effects on obesity (92–94). This highlights the complexity of microbial regulation in weight management.

The mechanisms by which gut microbiota influence obesity are multifaceted. They include (1) participating in energy homeostasis through the fermentation-derived production of short-chain fatty acids (SCFAs); (2) affecting nutrient absorption; (3) regulating lipoprotein lipase activity to promote fat storage; and (4) inducing low-grade inflammation and metabolic endotoxemia (83, 89, 95–98). It is particularly interesting that individual SCFAs—butyrate, propionate, and acetate—exert distinct and sometimes opposing metabolic effects. The relationship between microbial shifts in obesity and SCFA production is not straightforward (99–104). For example, the abundance of the anti-inflammatory, butyrate-producing bacterium *Faecalibacterium prausnitzii* may decrease in obese individuals, while certain Firmicutes members like *Lactobacillus reuteri* may increase (105–107). Secondly, microbial metabolites such as lipopolysaccharide (LPS) can increase intestinal permeability, leading to endotoxemia. This activates inflammatory pathways via Toll-like receptors (TLRs), resulting in systemic low-grade inflammation and insulin resistance (108, 109). This inflammatory state not only impairs insulin signaling but also exacerbates adipose tissue inflammation, further driving obesity and metabolic dysfunction (80, 110). Additionally, alterations in bile acid metabolism represent another key pathway. The gut microbiota can modulate the composition and metabolism of bile acids, thereby influencing lipid metabolism and insulin sensitivity (111, 112). Dietary patterns have been shown to shape the microbiota in ways that impact bile acid metabolism and, consequently, host metabolic health (111).

Although the F/B ratio was once widely considered a potential biomarker for obesity (113, 114), numerous studies have failed to consistently confirm its alteration. Methodological limitations, particularly insufficient sample sizes leading to low statistical power, are a major source of this heterogeneity. One analysis suggested that extremely large cohorts would be required to reliably detect the small differences in this ratio (115). Consequently, the current consensus is that the F/B ratio itself is not a robust marker of obesity-associated dysbiosis. Obesity is more likely linked to specific alterations at the genus or species level and an overall reduction in microbial diversity (116). Future research must look beyond phylum-level ratios to delve deeper into specific microbial functions and their interactions with the host.

In summary, the gut microbiota drives the development and progression of obesity and insulin resistance through diverse mechanisms affecting host energy metabolism and immunity. Understanding these mechanisms not only sheds light on the pathophysiology of obesity but also reveals potential therapeutic targets (79, 80, 117). Future studies should further explore strategies to modulate the gut microbiota for improved metabolic health, particularly in the prevention and management of obesity and its related disorders (111, 112).

## 5 Mechanisms underlying the beneficial effects of Urolithin A on obesity and related metabolic disorders

### 5.1 Activating adipose tissue thermogenesis and inducing white fat browning

UroA remodels adipose tissue function, promoting energy expenditure through thermogenic activation. The core mechanism involves activating the PGC-1α/UCP1 axis and stimulating mitochondrial biogenesis, thereby promoting thermogenesis in brown fat and inducing the browning of white fat. However, it should be noted that while these effects are robust in rodents, they appear to be limited and variable in humans due to species-specific differences in adipose tissue plasticity and browning capacity (118, 119). This process increases basal energy expenditure to counteract obesity (120).

**Figure 5:**
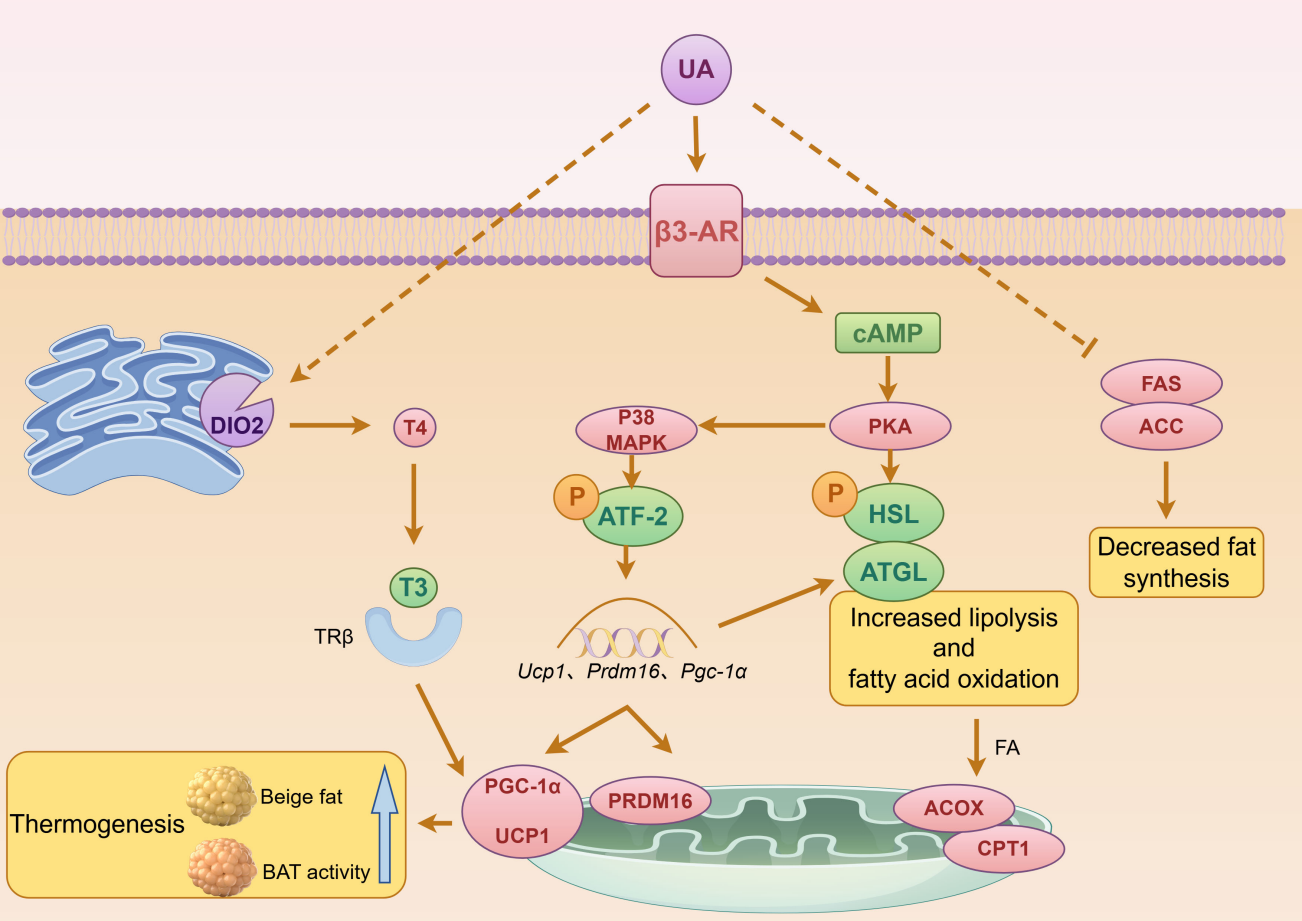
Mechanisms of Urolithin A-induced thermogenesis and metabolic regulation. Urolithin A (UA) activates β3-adrenergic receptor (β3-AR), triggering cAMP/PKA signaling to enhance lipolysis and fatty acid oxidation while suppressing fat synthesis. Concurrently, UA activates DIO2-mediated T4-to-T3 conversion, stimulating PGC-1α/UCP1 axis to promote thermogenesis in brown and beige adipose tissue.

The anti-obesity effect of UroA is mediated primarily by elevating triiodothyronine (T3) levels in brown adipose tissue (BAT) and inguinal fat depots. This effect depends on thyroid hormone regulation: blocking thyroid hormone synthesis abolishes the benefit, while supplementing with thyroxine (T4) restores it (120). UroA treatment enhances the expression and activity of type 2 iodothyronine deiodinase (DIO2), which converts the inactive prohormone T4 into the active hormone T3. This rise in T3 subsequently upregulates the expression of uncoupling protein 1 (UCP1) and peroxisome proliferator-activated receptor gamma coactivator 1-alpha (PGC-1α), promoting thyroid hormone-dependent energy expenditure. The final outcome is enhanced BAT thermogenesis and WAT browning (120)(**Figure 5**).

However, the dependence on thyroid hormone signaling raises important considerations regarding tissue-specific hormone metabolism. While UroA enhances peripheral DIO2 activity and local T3 production in adipose tissue, current evidence indicates that this effect remains compartmentalized with no significant disruption of systemic thyroid homeostasis in preclinical or short-term clinical studies (121). Notably, *in vitro* studies have actually identified potential anti-thyroid hormone receptor activities of UroA, suggesting complex endocrine interactions that warrant further investigation (122, 123). Patients with subclinical hyperthyroidism warrant particular attention, as they already exhibit suppressed thyroid-stimulating hormone (TSH) levels despite normal peripheral hormone concentrations; pharmacological stimulation of the thyroid axis in this population may exacerbate cardiovascular risks—including atrial fibrillation and coronary heart disease—and increase susceptibility to hip and vertebral fractures (124, 125). Furthermore, the long-term consequences of sustained AMPK-SIRT1-PGC-1α axis activation on hypothalamic-pituitary-thyroid (HPT) feedback mechanisms remain poorly characterized, and whether chronic UroA exposure could induce a state of functional thyrotoxicosis or trigger autoimmune thyroid responses requires careful investigation (126). Future clinical applications should monitor thyroid function parameters to confirm the absence of systemic effects in susceptible populations.

Despite these potential endocrine concerns, the mechanistic cascade underlying UroA-induced thermogenesis continues to highlight PGC-1α as a master regulator. Within this cascade, PGC-1α serves as a master regulator of mitochondrial biogenesis, playing a vital role in adipose tissue energy metabolism. AMPK and SIRT1 promote mitochondrial biogenesis and adipose tissue browning by regulating PGC-1α, thereby increasing energy expenditure and heat production (127, 128). Furthermore, fibroblast growth factor 21 (FGF21) also regulates UCP1 expression by elevating PGC-1α protein levels, promoting the browning and thermogenic capacity of white fat. This reinforces the central importance of the PGC-1α/UCP1 axis in adipose tissue remodeling (129). UCP1 itself is the core thermogenic protein in brown fat; its expression and activity directly determine the tissue’s heat-dissipating capacity. Other compounds, such as Tanshinone IIA and its derivatives, have been shown to promote white fat browning and brown fat activation via the AMPK-PGC-1α pathway, leading to increased UCP1 expression (130). Similarly, phospholipase A2 (PLA2G2A) enhances the thermogenic capacity of fat by boosting UCP1 expression and mitochondrial respiration (131).

In addition to the thyroid hormone axis, UroA can induce browning in 3T3-L1 white adipocytes by activating the β3-adrenergic receptor (β3-AR) and the p38 mitogen-activated protein kinase (MAPK)-dependent signaling pathway, thereby elevating thermogenic capacity (132) (**Figure 5**). At a concentration of 15 μM, UroA significantly upregulates beige-specific markers (Cd137, Cidea, Cited1, Tbx1, and Tmem26) and brown-fat-specific genes (Ppargc1a, Prdm16, and Ucp1), driving the conversion of 3T3-L1 white adipocytes toward a brown-like phenotype (132) (**Figure 5**). It should be noted that this concentration exceeds typical circulating levels observed in humans. Following oral administration of UroA or consumption of ellagitannin-rich foods, plasma concentrations in humans range from approximately 0.003 to 5.2 μM (27). Therefore, the 15 μM concentration employed in these *in vitro* studies represents a supraphysiological dose that may not be readily achievable in systemic circulation under normal dietary or standard supplemental conditions.

The anti-obesity effect of UroA may also involve modulation of the irisin signaling pathway. This hormone acts on adipose tissue via an endocrine route, inducing the differentiation of white adipose tissue (WAT) precursor cells into beige adipocytes. Characteristic changes include upregulated UCP1 expression, formation of multilocular lipid droplets, and enhanced mitochondrial biogenesis (133). Irisin further reinforces the beige adipocyte differentiation process by promoting mitochondrial proliferation within white adipocytes (53).

### 5.2 Regulation of lipid metabolism and adipogenesis

Research indicates that Urolithin A (UroA) and its congener urolithin B (UroB) exert potent anti-obesity effects in HFD-induced obese rodents. Following 4 weeks of intraperitoneal administration (2.5 mg/kg body weight, four times weekly) to rats rendered obese by 10 weeks of HFD feeding, UROA and UroB significantly reduced final body weight by approximately 19% and 21%, respectively, relative to vehicle-treated HFD controls (134). Both metabolites markedly attenuated visceral adipose tissue accumulation.

UroA exerts these effects through a dual metabolic mechanism that appears to operate independently of reduced caloric intake. Although pair-feeding controls were not explicitly included in the animal study, the magnitude of visceral adipose tissue reduction (35-44%) alongside significant increases in fecal lipid excretion and upregulation of hepatic fatty acid oxidation markers (PPARα) suggests a direct metabolic action rather than anorexia-induced weight loss (134, 135). Specifically, UroA promotes the oxidative breakdown of fatty acids and enhances fecal lipid excretion, while concomitantly suppressing *de novo* lipogenesis, thereby attenuating overall lipid accumulation (13, 136).

UroA orchestrates systemic lipid homeostasis through tissue-specific regulatory networks centered on AMPK activation in metabolically active organs, complemented by peripheral enzymatic inhibition. In hepatocytes and adipocytes, UroA functions as a metabolic switch by activating AMP-activated protein kinase (AMPK) (118, 119), which bifurcates into distinct effector arms: (i) catabolic enhancement via AMPK/ULK1-mediated lipophagy and PPARα-dependent β-oxidation (118, 119); and (ii) anabolic suppression via inhibition of lipogenic transcription factors (SREBP-1c) and their downstream targets (ACC/FAS); and (ii) transcriptional reprogramming via PPARα-mediated upregulation of fatty acid β-oxidation machinery and autophagy-mediated lipid droplet clearance (118, 119). Independent of these intracellular energy-sensing pathways, UroA exerts pre-absorptive inhibition of pancreatic lipase, reducing dietary lipid availability (137).

In adipose tissue, UroA attenuates lineage commitment and lipid storage capacity through transcriptional downregulation of the master adipogenic regulator PPARγ and its downstream target FABP4 (137). Notably, UroA exhibits the most potent anti-adipogenic activity among pomegranate metabolites in 3T3-L1 cells (137), an effect mechanistically linked to AMPK activation in human adipocytes (118).

In hepatic contexts, UroA restores fructose-impaired lipid homeostasis by activating the AMPK/ULK1 axis to stimulate lipophagy—the selective autophagic degradation of lipid droplets—thereby fueling mitochondrial β-oxidation (119). Concomitantly, UroA suppresses *de novo* lipogenesis via downregulation of ACC and fatty acid synthase (FAS) (119), effectively reprogramming hepatic lipid metabolism from storage to oxidation.

At the gastrointestinal interface, UroA inhibits pancreatic lipase activity with an IC_50_ of 0.032 mg/mL (137), representing a distinct pre-systemic mechanism that reduces dietary triglyceride hydrolysis and subsequent absorption, operating upstream of the cellular metabolic programs described above.

### 5.3 Remodeling the immune microenvironment and anti-inflammatory effects

UroA alleviates obesity-associated chronic inflammation primarily through macrophage remodeling. In obesity, adipose tissue expansion induces hypoxia and cellular stress, promoting the release of pro-inflammatory mediators such as free fatty acids and tumor necrosis factor-alpha (TNF-α). This microenvironment drives macrophage infiltration into adipose tissue and skews their polarization toward a pro-inflammatory (M1-like) phenotype (138). These M1-like macrophages secrete cytokines including interleukin-6 (IL-6) and IL-1β, exacerbating both local and systemic inflammation.

UroA counteracts this by modulating macrophage plasticity, shifting polarization from the insulin resistance-promoting M1-like state toward an anti-inflammatory, reparative M2-like phenotype—a transition confirmed in both *in vivo* and *in vitro* studies (139, 140). Macrophage activation is now recognized as a dynamic continuum rather than a binary switch (141), and UroA appears to steer this spectrum toward homeostatic resolution. In experimental models using peritoneal macrophages and bone marrow-derived macrophages (BMDMs), UroA pretreatment significantly downregulated the expression of M1-like markers (CD11c, TNF-α, IL-6, IL-1β, MCP-1) while upregulating M2-like markers (CH3L3, MGL2) (139).

This polarization shift is underpinned by UroA-mediated enhancement of mitochondrial oxidative metabolism, which provides the metabolic reprogramming required for anti-inflammatory phenotypes and facilitates inflammatory resolution (142). At the tissue level, UroA reduces pro-inflammatory factor expression in both liver and adipose tissue, coinciding with improved mitochondrial activity and biogenesis (139).

Within adipose tissue, UroA’s anti-inflammatory effects are multifaceted: it not only curbs macrophage infiltration but also reduces adipocyte hypertrophy and increases levels of the beneficial adipokine adiponectin (139). Given that adipose tissue inflammation is a pivotal driver of systemic insulin resistance, UroA’s modulation of mitochondrial activity and inflammation directly translates to improved insulin sensitivity.

Mechanistic studies show that the M1-like macrophage-dominated inflammatory response can impair insulin signaling in adipocytes by inhibiting key molecules like insulin receptor substrate-1 (IRS-1), ultimately contributing to insulin resistance and type 2 diabetes. Chronic inflammation also stimulates macrophages to secrete pro-fibrotic factors like transforming growth factor-beta (TGF-β), accelerating adipose tissue fibrosis and further compromising metabolic function. In line with this, UroA has been shown to effectively ameliorate high-fat/high-sugar (HF/HS) diet-induced insulin resistance in mice by directly enhancing insulin sensitivity in the liver and adipose tissue (143).

UroA’s benefits on glucose metabolism extend beyond inflammation. It upregulates the gene expression and secretion of both GLUT4 and adiponectin—two central players in enhancing insulin sensitivity and glucose uptake (137). In muscle tissue, UroA activates the PI3K/Akt and AMPK signaling pathways to promote GLUT4 translocation to the cell membrane, significantly boosting glucose uptake (144). Furthermore, by regulating autophagy and the AKT/mTOR pathway, UroA inhibits pancreatic β-cell apoptosis, thereby supporting insulin biosynthesis and secretion (145, 146).

UroA also targets digestive enzymes to modulate glucose homeostasis. It dose-dependently inhibits α-glucosidase (α-GLU) in the digestive tract, slowing carbohydrate absorption and helping to control postprandial blood glucose. Additionally, by inhibiting dipeptidyl peptidase-4 (DPP-4)—the enzyme that inactivates glucagon-like peptide-1 (GLP-1)—UroA prolongs GLP-1 activity, which in turn promotes glucose-dependent insulin secretion (137). An interesting metabolic shift is observed in macrophages, where UroA can reduce their glycolytic activity (147).

Enhanced mitochondrial activity is crucial for optimizing energy metabolism and alleviating obesity-related metabolic dysfunction. By optimizing mitochondrial function, UroA significantly increases energy metabolism efficiency, which concurrently elevates insulin sensitivity and suppresses inflammatory cascades. These synergistic actions collectively improve glucose intolerance and optimize whole-body glycemic control. This regulatory effect offers important protective benefits for maintaining systemic metabolic homeostasis under pathological conditions such as aging, obesity, and insulin resistance (139, 148).

### 5.4 Modulating gut microbiota and gut-axis communication

As a gut microbial metabolite, urolithin A (UroA) and its structural analogs have recently demonstrated considerable potential in regulating the gut microbiota and gut-organ axis communication. Studies show that UroA can influence the composition and function of the gut microbial community through various mechanisms, contributing significantly to metabolic health. Specifically, UroA and its analogs can suppress body weight gain in high-fat diet (HFD)-induced obese mice by through specific alterations in gut microbial composition, including documented enrichment of *Akkermansia* spp. and *Faecalibacterium prausnitzii* alongside contraction of lipopolysaccharide-producing *Proteobacteria*. This aligns with prior observations that individuals with obesity tend to have lower gut microbial diversity and richness compared to lean individuals (78, 149).

New experimental data further illustrate this point. In HFD-fed rats, UroA intervention resulted in a significantly lower Firmicutes-to-Bacteroidetes (F/B) ratio compared to untreated controls (p < 0.05). Relative to a normal diet (ND) group, the HFD group showed a 67.3% reduction in Bacteroidetes abundance and an 82.1% increase in Firmicutes. UroA treatment partially reversed these changes, restoring Bacteroidetes to 89.2% of the ND group level while reducing Firmicutes by 41.5% (150). Furthermore, both HFD feeding and UroA intervention significantly affected other microbial groups, such as Proteobacteria (p < 0.01). These findings suggest that UroA’s weight-reducing effects may be partly achieved by rebalancing the dynamic between Bacteroidetes and Firmicutes. However, given the documented inconsistency of F/B ratio associations with metabolic health (as discussed in Section 4.2.2), these changes should be interpreted with caution; the observed metabolic improvements more likely reflect concomitant shifts in lower taxonomic levels, such as increased abundance of *Akkermansia muciniphila* and enhanced SCFA production capacity, rather than phylum-level ratio shifts per se.

Beyond compositional shifts, UroA enhances gut barrier function by reversing high-fat-diet-induced alterations in microbial community structure. For instance, it can alleviate intestinal damage caused by environmental toxins like inorganic arsenic (151). This protective mechanism involves inhibiting oxidative stress and inflammatory markers, as well as preserving tight junction proteins in the gut epithelium, which collectively reduce intestinal permeability and prevent endotoxin translocation into the bloodstream (151). Additionally, UroA improves gut health by modulating specific microbial metabolic pathways, a function crucial for maintaining proper gut-axis communication (152).

UroA also exerts systemic metabolic benefits via gut-axis signaling. Research indicates that UroA can inhibit tumor progression by activating the autophagy-Hippo pathway and modulating the gut microbiota, suggesting that similar mechanisms might influence metabolic health (153). Moreover, by correcting gut dysbiosis and regulating microbial tryptophan metabolism, UroA activates the AhR/IL-22 axis, exerting anti-inflammatory effects in mouse models of colitis—a mechanism potentially relevant to obesity-related metabolic disorders (154).

Collectively, this evidence positions UroA, as a natural microbial metabolite, with promising potential to mitigate obesity and its associated metabolic disturbances by fine-tuning the gut ecosystem and gut-organ crosstalk. These insights provide a strong scientific foundation for developing novel intervention strategies against metabolic diseases.

## 6 From preclinical models to human clinical trials

### 6.1 Preclinical evidence for body weight regulation

Preclinical evidence indicates that UroA prevents diet-induced obesity via tissue-specific mechanisms. Although UroA confers systemic metabolic benefits—including enhanced mitochondrial function in skeletal muscle and modulation of gut microbiota composition—adiposity reduction is primarily driven by adipose tissue-specific thermogenic activation via thyroid hormone signaling, whereas muscle metabolic enhancement and gut microbiota remodeling serve as complementary pathways that support metabolic homeostasis without directly contributing to fat mass reduction (45, 155–157).

#### 6.1.1 Adipose tissue thermogenesis: primary driver of weight loss

Experimental evidence supports a causal link between UroA-induced thermogenesis and body weight reduction. Xia et al. demonstrated that in high-fat diet-fed mice, pharmacological blockade of thyroid hormone synthesis with propylthiouracil (PTU) completely abolished UroA-mediated brown adipose tissue activation, white fat browning, and the associated decrease in body weight (158). Exogenous thyroxine (T4) administration restored these metabolic effects, confirming that adipose tissue thermogenesis is necessary for UroA-induced weight loss. These findings demonstrate that the anti-obesity effects are strictly dependent on thyroid hormone signaling in adipose tissue, distinguishing direct thermogenic action from systemic metabolic improvements.

#### 6.1.2 Distinct mechanisms in muscle and gut microbiota

Work by Luan et al. demonstrated that UA treatment significantly enhanced muscle strength, exercise capacity, and mitochondrial respiratory capacity in dystrophic mouse models. Notably, this study focused on muscular dystrophy pathophysiology rather than obesity phenotypes, with primary endpoints centered on muscle function restoration rather than adiposity reduction. Although body composition was monitored by EchoMRI, the reported outcomes emphasized muscle-specific improvements (mitophagy activation, force generation, and fatigue resistance) distinct from systemic metabolic changes, supporting the concept that muscle metabolic benefits constitute a pathway separable from adipose tissue-mediated weight regulation (157).

Similarly, UroA-mediated gut microbiota remodeling contributes to metabolic health primarily by attenuating systemic inflammation and enhancing intestinal barrier function, rather than by directly modulating energy expenditure. Although the absence of UroA-producing bacteria (UM-0 phenotype) correlates with metabolic dysfunction, direct UroA supplementation circumvents the need for endogenous microbial biosynthesis, exerting anti-obesity effects through direct action on adipocytes, indicating that while UM-0 metabotype is associated with metabolic dysfunction, microbiota-independent UroA administration is sufficient to induce weight loss (45).

In summary, preclinical evidence supports a hierarchical model in which UroA reduces body weight primarily via adipocyte-autonomous thermogenesis dependent on thyroid hormone signaling, whereas alterations in muscle metabolism and gut microbiota composition appear to serve as complementary pathways that sustain metabolic homeostasis rather than directly contributing to fat mass reduction.

### 6.2 Promising findings from human clinical research

While large-scale clinical trials specifically targeting vascular function in obese populations are still underway, existing studies have already uncovered beneficial effects of Urolithin A (UroA) at multiple physiological levels. A 2024 clinical intervention study demonstrated that walnut supplementation (a dietary precursor of UroA) led to increased urinary UroA levels, which correlated significantly with reduced serum inflammatory markers. This, together with *in vitro* evidence of UroA-mediated suppression of pro-inflammatory cytokines, suggests UroA as a key bioactive metabolite that may improve the vascular milieu (159).

At the mechanistic level, a 2025 study provided novel evidence that UroA enhances mitophagy and restores intercellular communication via a calcium signaling pathway involving the inositol trisphosphate receptor (ITPR) and the mitochondrial calcium uniporter (MCU)—fundamental processes for maintaining vascular cell health (160). These key components of calcium signaling facilitate UroA-induced mitophagy, thereby improving mitochondrial function and organelle crosstalk (161, 162). In cardiomyocytes, where calcium signaling is crucial for mitochondrial homeostasis, UroA-mediated modulation of MCU activity has been shown to mitigate ischemia-reperfusion injury (162). Complementing these findings, an animal study directly demonstrated that UroA activation of the SIRT1 pathway ameliorates diabetes-induced cardiomyopathy and cardiac dysfunction (163). SIRT1, an NAD+-dependent deacetylase, confers cardioprotection by regulating autophagy and antioxidant responses (164, 165). For instance, SIRT1 promotes mitochondrial autophagy in cardiomyocytes by deacetylating transcription factors like FOXO1, thereby reducing oxidative stress and apoptosis (166, 167).

Investigations into exercise performance have yielded consistent results. An 8-week, double-blind, randomized controlled trial involving male resistance-trained athletes showed that UroA supplementation significantly improved muscle endurance and strength while also ameliorating markers of oxidative stress and inflammation (168). This aligns with prior observations on the positive impact of certain supplements on exercise performance and physiological recovery.

Collectively, this multifaceted evidence builds a coherent model in which the benefits of UroA span from dietary intake and anti-inflammatory effects to cellular function and organ protection. The consistent findings from both human and mechanistic studies underscore UroA’s potential as a multi-level therapeutic agent, warranting further exploration in clinical nutrition and metabolic health strategies.

## 7 Conclusion

This review has comprehensively examined the conversion pathway from dietary ellagitannins (ETs) to Urolithin A (UroA) and its multifaceted mechanisms of action in obesity intervention. UroA is not directly present in foods but is the terminal bioactive metabolite of ETs (e.g., punicalagin in pomegranate), produced via gut microbial metabolism. This transformation exhibits significant inter-individual variation, regulated by multiple factors including specific microbial community composition, age, and body mass status.

In combating obesity and related metabolic disorders, UroA engages a network of synergistic pathways. Its core mechanisms include: (1) activating thermogenesis in brown adipose tissue and inducing browning of white adipose tissue, primarily by modulating thyroid hormone activity and the PGC-1α/UCP1 signaling axis to convert energy storage depots into energy-consuming organs; (2) bidirectionally regulating lipid metabolism by promoting fatty acid β-oxidation and autophagic breakdown while simultaneously inhibiting the expression of key lipogenic genes (e.g., ACC, FAS, PPARγ) and the digestion/absorption of dietary fats; (3) remodeling the immunometabolic microenvironment by driving macrophage polarization from the pro-inflammatory M1-like phenotype toward the anti-inflammatory M2-like phenotype, thereby alleviating chronic low-grade inflammation and improving systemic insulin sensitivity and glucose homeostasis; (4) modulating gut microbial composition, improving microbial diversity indices compromised by high-fat diet feeding, and enhancing intestinal barrier function.

Robust preclinical evidence strongly supports the metabolic benefits of UroA, while preliminary human studies have observed its association with reduced inflammation, improved vascular function, and enhanced exercise performance. In summary, UroA, as a natural metabolite derived from diet-microbiota interactions, represents a promising multitarget intervention strategy for the prevention and management of obesity and its complications. While preclinical studies demonstrate robust metabolic benefits, human evidence remains limited to small-scale observations of surrogate endpoints such as inflammatory markers and vascular function. Thus, UroA currently represents a mechanistically promising but clinically unvalidated candidate for obesity management. Its potential therapeutic value depends on integrated regulation of host metabolism and inflammation, though translation to evidence-based practice requires substantially more rigorous investigation.

## 8 Discussion

The research journey of Urolithin A (UroA) vividly illustrates the intricate complexity of the “diet-microbiome-host health” axis. The evidence synthesized in this review indicates that UroA’s biological effects are fundamentally rooted in its unique origin as a microbiota-derived metabolite and its multi-target mode of action. This very nature, however, raises several important scientific questions for future exploration.

First, the pronounced interindividual variation in UroA’s efficacy presents a central challenge for its clinical translation. The widespread prevalence of the three urolithin metabotypes (UM-A, UM-B, and UM-0) means that simply increasing dietary intake of ellagitannins (ETs) may offer minimal benefit to the approximately one-third of individuals (UM-0) who cannot produce UroA (32, 43). This poses a key question for precision nutrition: should future strategies involve stratifying populations based on gut microbiota profiling, with direct UroA or precursor probiotic supplementation for UM-0 individuals? Importantly, UM phenotypes are not static; their correlation with age and obesity status suggests that microbial metabolic capability may itself be a dynamic biomarker of metabolic health, rather than a simple causal determinant (32, 169).

Second, UroA exhibits a “systems biology”-like network of synergistic actions rather than targeting a single pathway. Its mechanisms—from activating adipose tissue thermogenesis and modulating lipid anabolism/catabolism to exerting anti-inflammatory effects and regulating gut microbiota—are interconnected, forming a coordinated defense against energy imbalance. For instance, the M2-like macrophages induced by UroA not only resolve inflammation but may also participate in tissue remodeling and metabolic regulation through secreted factors. Conversely, the improvement in gut microbiota is both a result of UroA’s action and a potential modulator of its ongoing production and efficacy. While this pleiotropy is a strength, it also complicates the identification of its primary molecular target.

Third, the translation from compelling preclinical models to human applications requires careful consideration. Despite encouraging animal data, large-scale, long-term human intervention trials directly validating UroA’s weight-loss effects remain scarce. Existing human studies have primarily focused on observational correlations, safety, or surrogate endpoints like inflammatory markers. The effective dose, optimal intervention duration, and long-term safety of UroA across different populations (e.g., varying UM phenotypes, degrees of obesity, age groups) must be confirmed through well-designed randomized controlled trials (44, 45, 120, 136). Furthermore, the potential synergistic effects of combining UroA with other metabolic interventions, such as dietary control or exercise, warrant investigation.

Moreover, critical pharmacological parameters—including bioavailability variations across metabotypes and long-term safety profiles—remain undefined. Whether direct UroA supplementation can effectively bypass the metabolic limitations of UM-0 individuals requires empirical validation. Potential interactions with existing metabolic medications and the durability of benefits after supplementation cessation also represent key unknowns. These limitations underscore that UroA remains an experimental compound; claims of therapeutic utility currently exceed the available clinical evidence.

Finally, research into UroA’s mechanisms has deepened our understanding of adipose tissue plasticity and immunometabolism. UroA is more than a “fat-burning” molecule; it acts as a potential metabolic-immune modulator based primarily on preclinical evidence. Its pathway of improving insulin resistance via macrophage polarization provides a concrete molecular case study for understanding obesity-associated chronic inflammation. Simultaneously, its ability to reshape the gut ecosystem extends the battlefield for metabolic health to the digestive tract, emphasizing that maintaining systemic metabolic homeostasis requires sophisticated cross-organ communication.

In conclusion, while UroA provides valuable insights into diet–microbiota–host interactions, it currently remains an experimental metabolite with robust mechanistic support but limited clinical validation. Future work should prioritize addressing these limitations—particularly the generation of rigorous clinical efficacy data—before UroA can be considered a viable component of evidence-based obesity management (170).
